# Relationship between periodontitis and systemic diseases: A bibliometric and visual study

**DOI:** 10.1111/prd.12621

**Published:** 2025-01-08

**Authors:** Gaetano Isola, Alessandro Polizzi, Sara Serra, Mattia Boato, Anton Sculean

**Affiliations:** ^1^ Department of General Surgery and Medical‐Surgical Specialities, Unit of Periodontology University of Catania Catania Italy; ^2^ Department of Periodontology University of Bern Bern Switzerland

**Keywords:** association, bibliometric analysis, CiteSpace, clinical trials, co‐occurrence mapping, correlation, host response, inflammation, microbiota, periodontitis, review, systemic diseases

## Abstract

To provide a comprehensive and updated mapping of observational studies assessing the relationship between periodontitis and systemic diseases through a bibliometric and visual analysis. A search was conducted using the Web of Science database, covering the period 1989 to 2024. The Medical Subject Headings (MeSH) from the US National Library of Medicine was used to categorize systemic conditions, focusing on terms unrelated to stomatognathic diseases. The analysis included keyword co‐occurrence mapping, co‐authorship, bibliographic coupling, and co‐citation analysis. Quality indicators such as silhouette score, modularity, and centrality were considered to assess the network's quality. The research strategy identified 6106 records, of which 1519 met the inclusion criteria. The analysis revealed that 46.73% of the literature on the topic was published in the last 5 years and that the annual publication trend peaked in 2023. Nutritional & Metabolic Diseases (*n* = 398), Cardiovascular Diseases (*n* = 335), Female Urogenital Diseases & Pregnancy Complications (*n* = 244), and Musculoskeletal Diseases (*n* = 182) were the most representative categories of systemic diseases associated with periodontitis. The most co‐cited journals on the topic were the Journal of Periodontology (*n* = 1412), the Journal of Clinical Periodontology (*n* = 1343), the Journal of Dental Research (*n* = 940), and Periodontology 2000 (*n* = 849). The USA, China, Brazil, and Sweden were the countries that contributed the most to the number of publications. The analysis conducted in the present study revealed a growing trend of observational studies evaluating the association between periodontitis and systemic diseases, highlighting the negative impact of periodontitis on a plethora of systemic conditions and a rising translational interest in this relationship. With an aging population, periodontitis is expected to affect a growing number of people in the coming decades, presenting significant challenges to public health. Improved knowledge is, therefore, essential to enable more comprehensive care, preventive strategies, and optimal oral health for patients with periodontitis.

## 1 INTRODUCTION

Periodontitis is a chronic, multifactorial inflammatory disease initiated by the accumulation of a dysbiotic biofilm on tooth surfaces followed by an immune‐inflammatory host response^1^, ^2^ that could result if not preventively diagnosed^3^, ^4^ and adequately treated,^5^, ^6^ in a progressive periodontal tissue destruction and tooth loss^7^ negatively affecting the overall quality of life.^8^ The global prevalence of mild forms of periodontitis has been estimated to be around 62% in the worldwide population,^9^ with severe forms of the disease affecting approximately 23.6% of them, making periodontitis the seventh most common disease in humans.^9^, ^10^ Over the past decades, a substantial body of evidence linked periodontitis to systemic conditions, mainly related to aging, impaired life, and also premature death.^11^, ^12^ In particular, in the last few decades, periodontitis was strictly associated with several systemic diseases^13^, ^14^ such as cardiovascular diseases,^15^, ^16^ cancer,^17^, ^18^ rheumatic disease,^5^, ^19^ diabetes and obesity,^20^ Alzheimer's disease,^21^, ^22^ and chronic lower respiratory diseases^23^ and increased risk of peri‐implantitis,^24^ all conditions reported with severe illness and death in the US report of the National Center for Health Statistics of the Centers for Disease Control and Prevention.^25^

In this regard, the 2012 European Federation of Periodontology—American Academy of Periodontology (EFP‐AAP) joint workshop, aimed at further evaluating the potential bidirectional relationship between periodontitis and various systemic diseases and conditions, highlighted the significant health, social and economic burdens that periodontitis reflects and contributes to global systemic health and social inequalities; the workshop identified, at the same time, the diagnosis and treatment of periodontitis as a strong influential factor in reducing the risk of developing a large number of associated systemic diseases, leading to improved overall health outcomes.^26^, ^27^, ^28^, ^29^, ^30^, ^31^, ^32^

Furthermore, a number of reports have highlighted the importance of oral health and disease in systemic conditions. The study conducted by Jepsen et al.,^33^ as a result of the consensus report of the 2017 World Workshop on the Classification of Periodontal and Peri‐Implant Diseases and Conditions that updated the old classification of periodontal manifestations and conditions affecting the periodontal apparatus, concluded highlighting the importance of periodontitis as a negative stimulus of systemic diseases. In agreement, Genco and Sanz^13^ reported that the field of periodontal medicine aimed at assessing the systemic impact of periodontitis is highly dynamic, with new disease associations appearing in rapid succession as a consequence of the exponential increase in scholarly publications, some of them with uncertain clear conclusions.

Given the vast and rapidly expanding body of literature, in the last few years, several methodological tools have been developed in order to better determine the evidence from studies in the literature.

In this regard, bibliometric and visual analysis studies, which involve the quantitative assessment of publications, citations, keyword co‐occurrence mapping, and research trends,^34^ have been demonstrated as advisable and supporting methods for identifying core research or authors, as well as their relationship, by covering all the publications related to a given topic or field.^35^, ^36^ In this regard, certain evidence aimed at analyzing the bibliometric mapping of clinical trial registers^37^ reported an increased research activity in periodontal medicine since the early 2000s. However, another preliminary bibliometric review that identified the most‐cited publications linking periodontitis to systemic conditions^38^ provided an important increased trend regarding the impact of periodontitis on systemic health but concluded that articles related to the several systemic manifestations of periodontal diseases were uncommon among the most‐cited publications, reflecting that more research on this topic is still required.

For these reasons, based on the above‐mentioned references, the present study was designed to provide an updated map of the current comprehensive state of knowledge related to observational studies linking periodontitis to systemic diseases using a bibliometric and visual analytic approach in order to further understand the associations between periodontitis and systemic diseases in order to better clarify and evaluate the possible significance of this relationship for both oral and systemic human health.

## 2 MATERIALS AND METHODS

### 2.1 Search strategy and categorization of systemic conditions

For the present analysis, the a search query was performed in July 2024 (7‐22‐2024) on the Web of Science (WoS) Core Collection without timeline restrictions, which included the following terms: “Periodontitis AND (bacteremia OR infection* OR virus OR cancer OR carcinoma OR neoplasm* OR osteoporosis OR *arthritis OR spondyl* OR gastr* OR esophag* OR liver OR respiratory OR pulmonary OR pneumonia OR chronic obstructive OR cerebrovascular OR Alzheimer OR dementia OR eye OR erectile dysfunction OR infertility OR chronic renal OR kidney OR pregnancy OR menopause OR premature birth OR pre‐eclampsia OR cardiovascular OR hypertension OR coronary heart OR myocardial infarction OR angina pectoris OR stroke OR atherosclerosis OR endothelial OR ischemic heart OR vascular OR arter* OR anemia OR autoimmune OR lupus erythematosus OR systemic sclerosis OR Sjogren OR diabetes mellitus OR metabolic OR obesity OR dyslipid* OR hyperlipid* OR vitamin D OR dyspepsia OR depression OR stress OR anxiety OR transplantation OR systemic disease*) AND (correlation OR association).” The inclusion criteria were (1) observational studies linking periodontitis to systemic conditions; (2) prospective, retrospective, or cross‐sectional study design; (3) English publication. The exclusion criteria were (1) observational studies linking periodontitis to other stomatognathic conditions; (2) narrative and systematic reviews; (3) interventional studies; (4) animal studies; and (5) opinion articles, editorials, conference reports, and surveys.

For the search analysis, the term “periodontitis” was employed in agreement with the 2017 classification of periodontal and peri‐implant diseases and conditions.^7^

The 2015 Medical Subject Headings (MeSH) from the US National Library of Medicine were chosen to elaborate the search strategy and define and categorize systemic diseases.^37^, ^39^ MeSH, a controlled vocabulary thesaurus used for PubMed article indexing (http://www.ncbi.nlm.nih.gov/mesh), has sixteen primary branches (A through N + V + Z), which results from a hierarchical categorization that produces more specialized words (taxa). Any MeSH word (taxon) not mentioned in [C07] (Stomatognathic disease) was categorized as a systemic disease.^37^ For categorization, if the MeSH term search found a sub‐branch of a specific disease, the primary domain of related disease manifestation was chosen to avoid redundancy in the search.

### 2.2 Study selection and data extraction

Two independent researchers (S.S., M.B.) screened the records for titles and abstracts, and the studies that didn't fit the inclusion criteria were discarded. The degree of agreement among reviewers was evaluated using Cohen's kappa agreement, which was 78.3%, denoting a good agreement. Conflicts among the reviewers on the ultimate selection of some articles, when present, were solved by a third reviewer (G.I.). The total number of citations and publications, the WoS categories and themes, journals, nations, keywords, authors, affiliations, and funding organizations were obtained from the WoS database.

### 2.3 Data processing and visual analysis

The final collection of included literature was loaded into CiteSpace 6.3.R2 (Dressel University, PA, USA) in plain text format to perform a bibliometric analysis evaluating ranks, centrality metrics, co‐occurrence, and clustering of keywords for authors, countries, and organizations. CiteSpace was created to visualize advancing knowledge areas and new trends. In the data processing, both manual and computerized analyses were performed. To obtain a reasonable period of analysis, the final program parameters for the bibliometric analysis set were “Time period = 1989–2024,” “Year Slice = 1,” “g‐index = 25,” and “Top N% = 10.” All the other parameters were selected to default.

VOSviewer 1.6.19.0 (Leiden University, Holland), which enables the creation of bibliometric visual networks and shows the relationships between different entities like publications, keywords, institutions, and researchers, was used to facilitate co‐authorship analysis, bibliographic coupling, and co‐citation production. To analyze and visualize the co‐occurrence networks of keywords, journals, authors, countries, and partnerships, the bibliometric analysis was carried out using the suggestions listed in the VOSviewer user manual.^40^

To highlight research trends based on the systemic conditions analyzed over the years, a connectogram was used to link the fraction of registrations by [Diseases‐C] sub‐branches to the study publishing years and a circular phylogenetic‐like tree to map visually systemic conditions and their relative sub‐branches that may be connected to periodontitis using the MeSH terms. A connectogram and phylogenetic‐like tree were created using Flourish diagram (https://app.flourish.studio/@daan/chord‐diagram).

### 2.4 Data analysis and indicators

Most of the study's findings were displayed as percentages and numbers along with graphic network maps. Basic literature analysis, clustering, and burst analysis were performed using CiteSpace to find recurring terms. Impact networks (journals and references), contribution and cooperation networks (authors, countries, and institutions), and keyword analysis (cluster) were applied to the analyses. The top 10 terms that had the greatest bursts of citations were assessed. Three criteria were also used to evaluate the output's quality: silhouette, modularity, and centrality. Centrality computes the shortest paths between each pair of nodes in the network to assess the importance of a node, particularly one that serves as a bridge or a central position in a cluster.^41^ By clustering strongly associated nodes together, keyword clustering was able to highlight relevant subject topics and their evolution across time.^42^ Modularity or Q scores, which vary from 0 to 1, were used to determine the quality of the cluster division in the network and a network with a Q score of more than 0.3 was considered well‐organized. Lastly, the silhouette score (S score), which has a range of −1 to +1, was used to assess the quality of the clustering arrangement. A network homogenous, satisfactory, or very reliable was denoted if its S score was more than 0.3, 0.5, or 0.7.^43^

## 3 RESULTS

**FIGURE 1:**
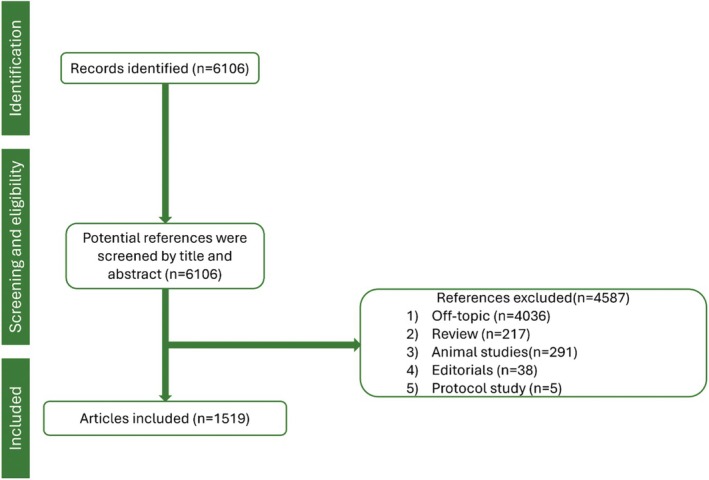
Flow chart of the study.

The research strategy led to the identification of 6106 records in the WoS database. After the screening by title and abstract, 4587 records were excluded because they were not directly related to the study's primary aim (*n* = 4036), for review design (*n* = 217), animal studies (*n* = 291), editorials (*n* = 38), and protocol studies (*n* = 5). Finally, 1519 articles met the inclusion criteria and were included in the present bibliometric analysis (Figure 1).

### 3.1 Annual publication trend

**FIGURE 2:**
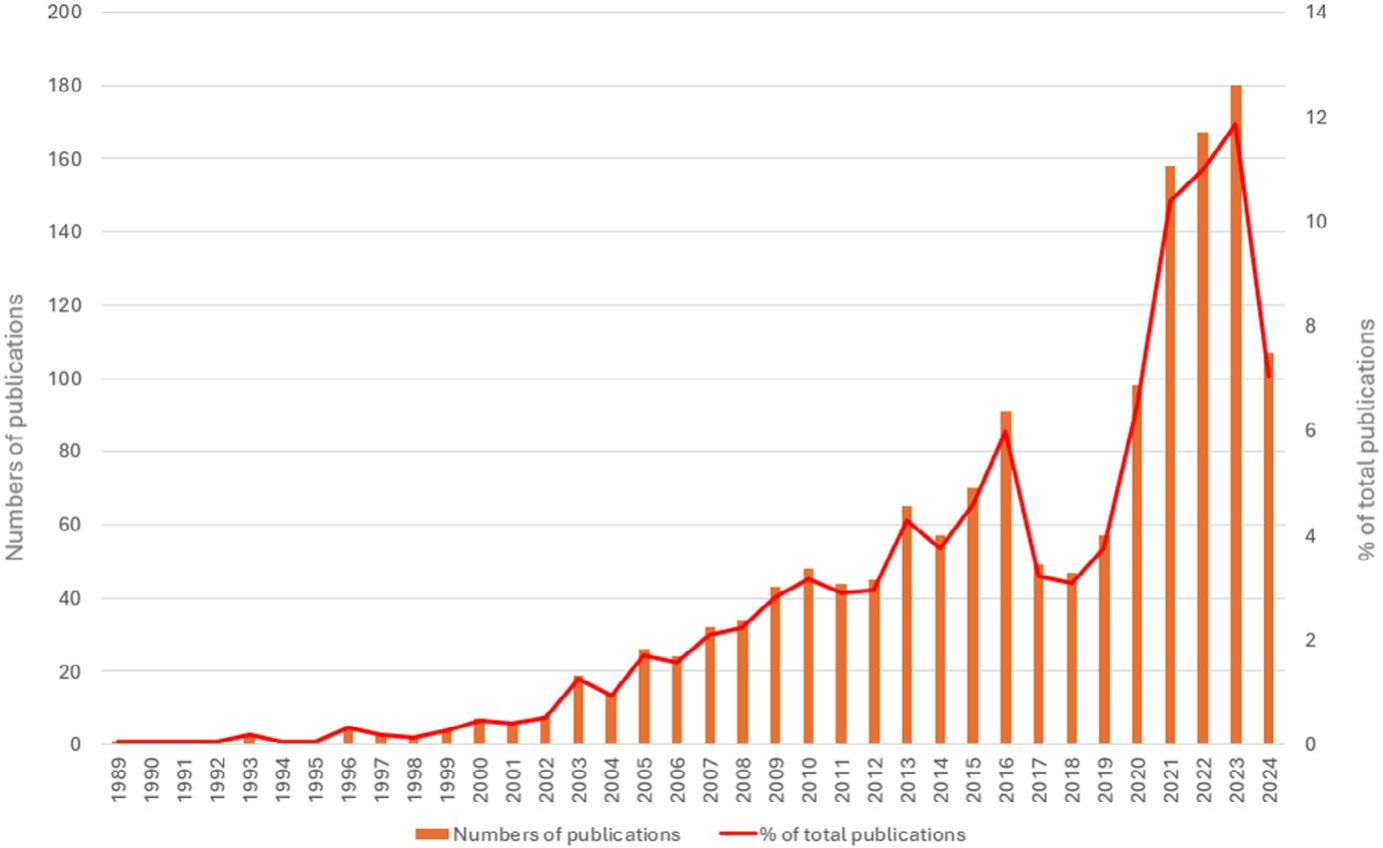
Publication trend from 1989 to 2024. The vertical bars indicate the absolute number of publications, whereas the red line represents the percentage proportion.

The annual publication trend is shown in Figure 2. The publication peak was reached in 2023 with 180 articles (11.85% of the total publications). Moreover, despite a gradual increase in publications related to the association between periodontitis and systemic diseases over the years, articles decreased in the 3 years 2017–2019 with a notable increase in the subsequent years. In fact, in the last 5 years, a total of 710 articles (46.73% of the total) were published on the topic. In the publication trend for 2024, it should be taken into account that the research is updated to 22th July and that only after the end of the year will it be possible to have a definitive picture of the current year's trend.

### 3.2 Journal analysis

As reported in Table 1, the top 5 most influential journals for the number of co‐citations of the association between periodontitis and systemic diseases were the *Journal of Periodontology* (counts = 1412), the *Journal of Clinical Periodontology* (counts = 1343), the *Journal of Dental Research* (counts = 940), *Periodontology 2000* (counts = 849), and the *Journal of Periodontal Research* (counts = 679). Although the dentistry category was the one with the most published articles (counts = 794), a substantial contribution also came from categories related to general medicine, public health, multidisciplinary sciences, endocrinology, immunology, and rheumatology, and vascular diseases. The most impactful WoS categories have been listed in Table S1.

**TABLE 1:** Most co‐cited journals for the association between periodontitis and systemic diseases. Year, year of the first published article in the related journal.

No	Count	Centrality	Year	WoS Category	Cited journals
1	1412	0.00	1989	Dentistry, Oral Surgery & Medicine	*Journal of Periodontology*
2	1343	0.00	1989	Dentistry, Oral Surgery & Medicine	*Journal of Clinical Periodontology*
3	940	0.00	1989	Dentistry, Oral Surgery & Medicine	*Journal of Dental Research*
4	849	0.00	1999	Dentistry, Oral Surgery & Medicine	*Periodontology 2000*
5	679	0.00	1991	Dentistry, Oral Surgery & Medicine	*Journal of Periodontal Research*
6	540	0.00	1999	Dentistry, Oral Surgery & Medicine	*Annals of Periodontology*
7	497	0.00	1991	Medicine, General & Internal	*Lancet*
8	415	0.00	2012	Multidisciplinary Sciences	*PLOS ONE*
9	403	0.00	1990	Medicine, General & Internal	*New England Journal of Medicine*
10	400	0.00	1993	Cardiac & Cardiovascular Systems	*Circulation*

### 3.3 Reference analysis

The top 10 articles for the number of citations are presented in Table 2. The most‐cited article^44^ reached 736 citations. Moreover, the two top‐cited articles were prospective studies with over 9000 included patients.^44^, ^45^

**TABLE 2:** Top 10 references for number of citations.

No	Citations	Article	Year	Author
1	736	Dental disease and risk of coronary heart disease and mortality	1993	De Stefano et al.
2	349	Periodontal disease and risk of cerebrovascular disease: the first national health and nutrition examination survey and its follow‐up study	2000	Wu et al.
3	346	Periodontal disease and the oral microbiota in new‐onset rheumatoid arthritis	2012	Scher et al.
4	337	Periodontal infection and preterm birth: results of a prospective study	2001	Jeffcoat et al.
5	333	Glycemic control of type 2 diabetes and severe periodontal disease in the US adult population	2002	Tsai et al.
6	315	Relationship of periodontal disease to carotid artery intima‐media wall thickness: the atherosclerosis risk in communities (ARIC) study	2001	Beck et al.
7	314	Relationship between periodontal disease, tooth loss, and carotid artery plaque: the Oral Infections and Vascular Disease Epidemiology Study (INVEST)	2003	Desvarieux et al.
8	304	Periodontal disease and coronary heart disease risk	2000	Hujoel et al.
9	283	Association of periodontal disease and tooth loss with rheumatoid arthritis in the US population	2008	De Pablo et al.
10	282	Poor oral hygiene as a risk factor for infective endocarditis–related bacteremia	2009	Lockhart et al.

### 3.4 Authors, Countries, and Institution analysis

**FIGURE 3:**
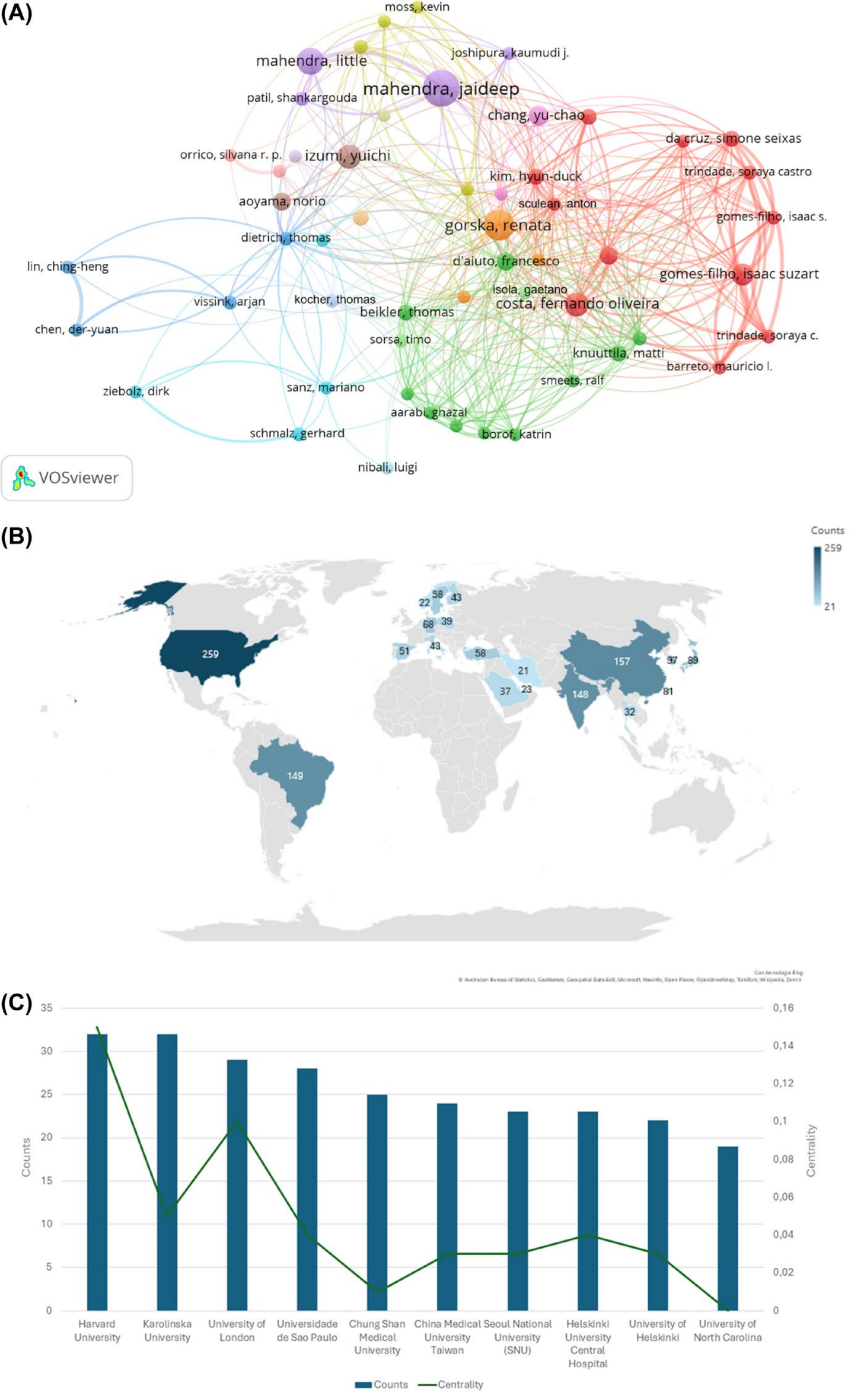
(A) Authors who cited each other the most number of times; (B) Graphic representation of the top nations for the number of published articles on the association between periodontitis and systemic conditions; (C) Publication trend per Institutions.

Table S2 lists the top 10 authors for the number of published articles related to the connections between periodontitis and systemic conditions. The author Jaideep Mahendra published 20 articles on the topic, whereas Renata Gorska and Little Mahendra published 17 and 15 articles, respectively. Interestingly, there was no significant association between the number of articles and received citations; Francesco D'Aiuto, with 10 published articles on the topic, received the most number of citations (counts = 658) compared to the other top 10 authors. Figure 3A shows a graphic representation of the cited authors who cited each other most of the time.

The most impactful nations for the number of published articles have been reported in Figure 3B. In particular, the USA is the first country in the world with a count of 259 papers and a high centrality (0.53), followed by China and Brazil with 157 and 149 articles and 0.05 and 0.03 centralities, respectively.

Figure 3C shows the publication trend related to the involvement of Institutions. In particular, Harvard University and Karolinska University were the first institutions for the number of published papers on the topic, with 32 articles each, followed by the University of London, Universidade de Sao Paulo, and Chung Shan Medical University with 29, 28, and 25 published papers, respectively. Moreover, Harvard University and the University of London showed the highest centrality scores (0.15 and 0.10, respectively), indicating a high degree of collaboration with other institutions.

### 3.5 Classification of systemic conditions

**FIGURE 4:**
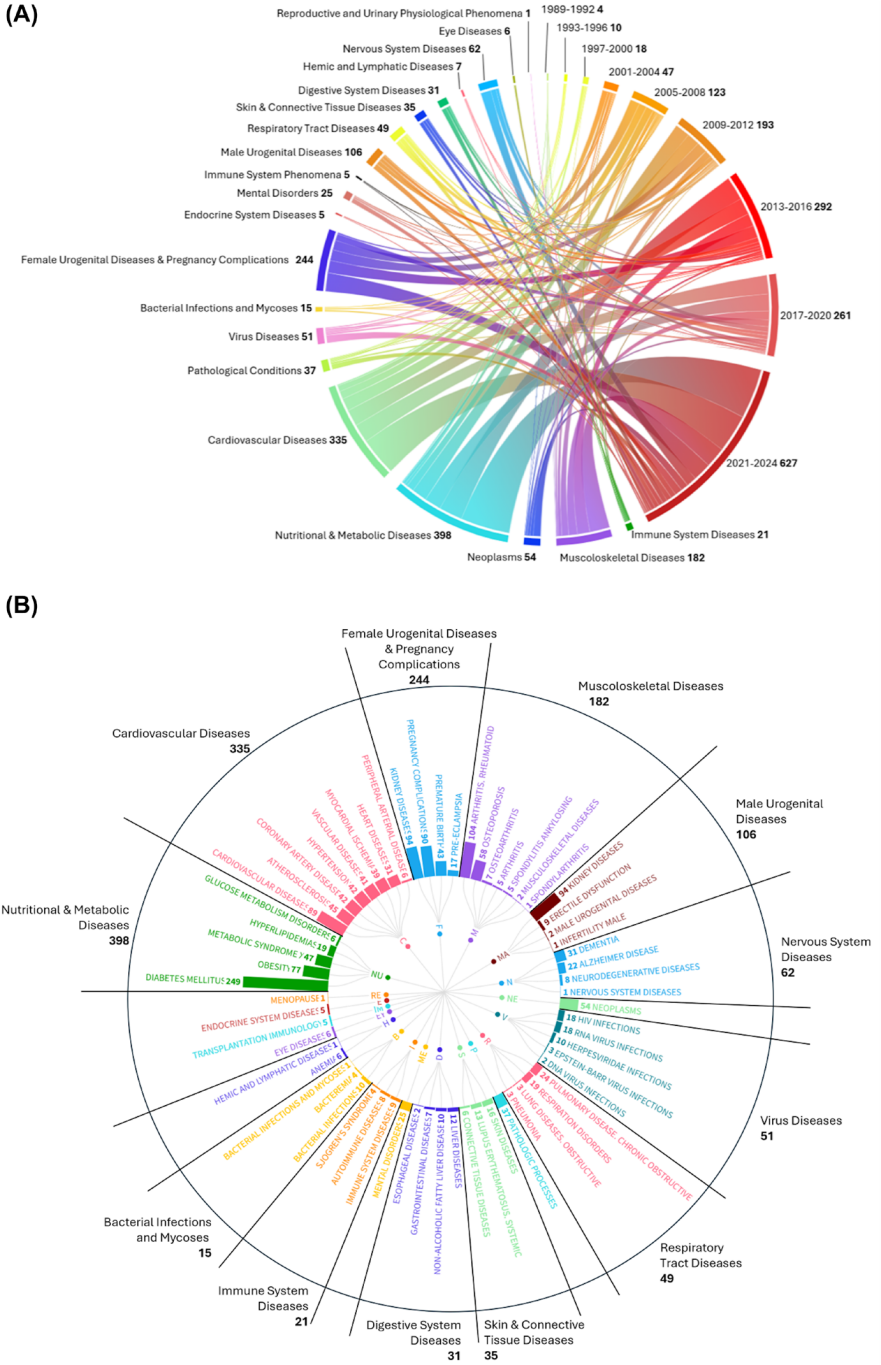
(A) Chord diagram of the temporal evolution of studies related to the association of periodontitis with systemic conditions. This connectogram shows the proportion of articles related to each sub‐branches of the MeSH classification “Diseases” [C] and “Phenomena and Processes” [G], linked to the respective years of publication grouped in 9 periods: 1989–1992, 1993–1996, 1997–2000, 2001–2004, 2005–2008, 2009–2012, 2013–2016, 2017–2020, 2021–2024. (B) Phylogenetic‐like tree of systemic conditions that have been supposed to be related to periodontitis. For each relevant systemic condition, subcategories were reported according to the hierarchical tree structure of the MeSH classification.

Figure 4A shows a chord diagram of the temporal evolution of studies related to the association of periodontitis with systemic conditions, according to the MeSH classification. The MeSH sub‐branches have been linked to the respective years of publication grouped in 9 periods: 1989–1992 (*n* = 4 articles), 1993–1996 (*n* = 10 articles), 1997–2000 (*n* = 18 articles), 2001–2004 (*n* = 47 articles), 2005–2008 (*n* = 123 articles), 2009–2012 (*n* = 193 articles), 2013–2016 (*n* = 292 articles), 2017–2020 (*n* = 261 articles), 2021–2024 (*n* = 627 articles). A progressively greater number of publications stands out, with the most published papers on the topic in the last 4 years. Moreover, Figure 4B presents a phylogenetic‐like tree of systemic conditions that have been supposed to be related to periodontitis. More specifically, Nutritional & Metabolic Diseases were the most representative category (*n* articles = 398), followed by Cardiovascular Diseases (*n* = 335), Female Urogenital Diseases & Pregnancy Complications (*n* = 244), and Musculoskeletal Diseases (*n* = 182). For each relevant systemic condition, subcategories were reported according to the hierarchical tree structure of the MeSH classification. Diabetes mellitus was the most studied systemic condition (*n* = 249), followed by rheumatoid arthritis (*n* = 104), kidney diseases (male and female *n* = 94, respectively) and cardiovascular diseases (*n* = 89).

### 3.6 Keyword analysis

#### 3.6.1 Keywords co‐occurrence

**FIGURE 5:**
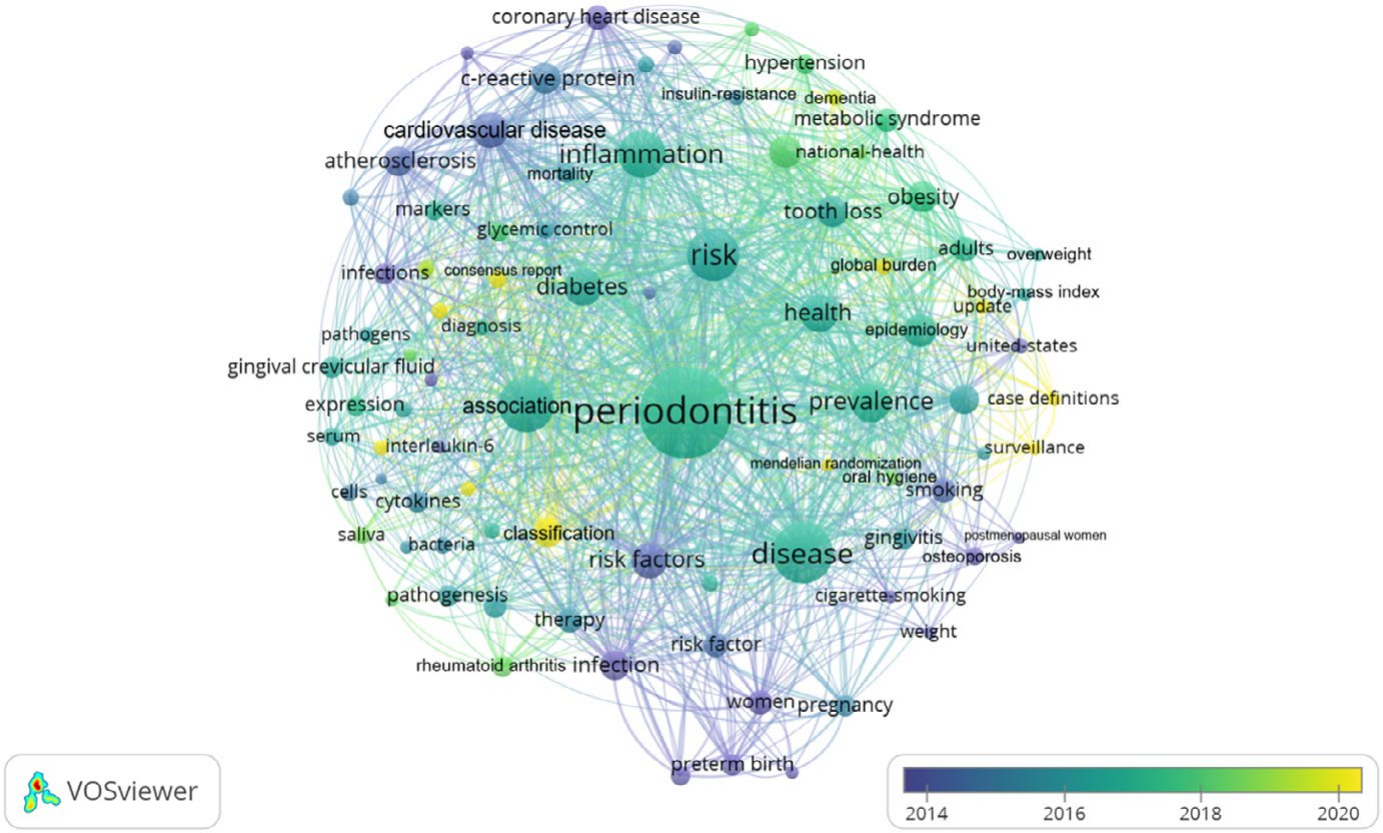
The co‐occurrences of the top 86 keywords with at least 25 apparitions.

Figure 5 presents the co‐occurrences of the top 86 keywords with at least 25 apparitions. In particular, the top‐occurring keyword was “periodontitis” with 1241 occurrences, followed by the terms “disease” (*n* = 552), “association” (*n* = 484), “risk” (*n* = 407), “inflammation” (*n* = 348), and “prevalence” (*n* = 236), all keywords are in accordance with the present bibliometric analysis.

#### 3.6.2 Keywords with citation burst

**FIGURE 6:**
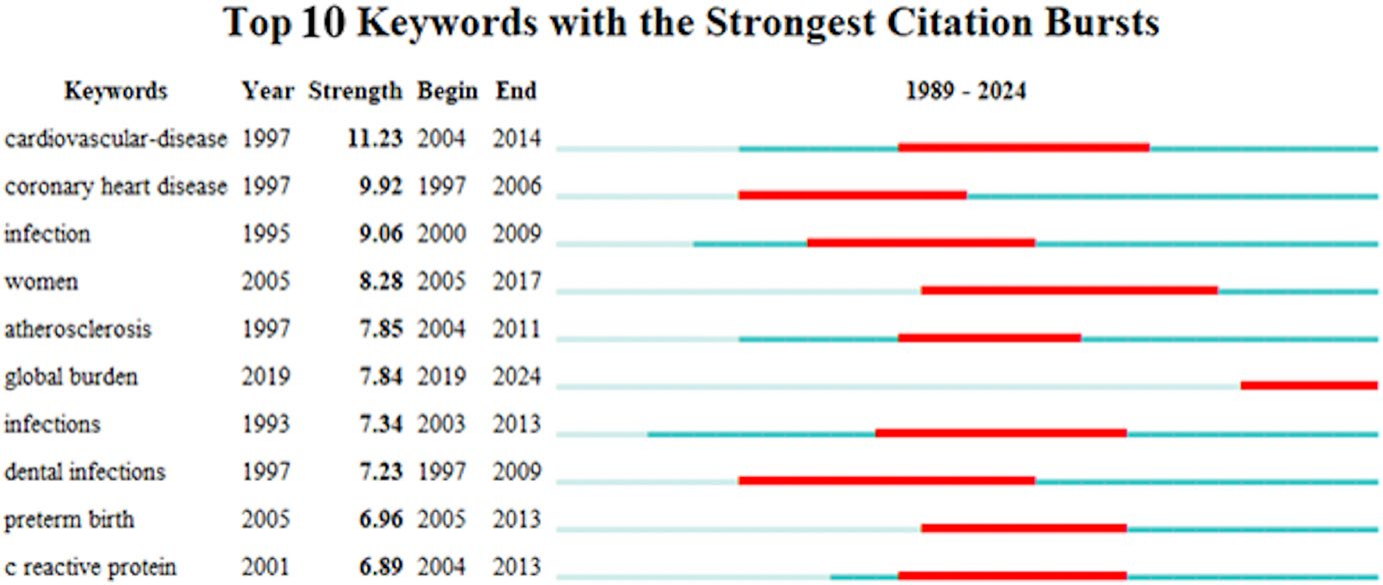
The top 10 keywords with the strongest bursts of citations; years with more frequent occurrences are indicated by the red line, and years with fewer appearances are indicated by the green line.

Citation burst keywords illustrate the research hotspots in a certain field of study at various times by showing which keywords have a high citation rate during those periods.^46^ Figure 6 shows the top 10 keywords with the strongest citation bursts. The top classified keywords in terms of strength were cardiovascular‐disease (strength = 11.23), coronary heart disease (strength = 9.92), infection (strength = 9.06), and women (strength = 8.28). Frequent occurrences have characterized cardiovascular‐disease and coronary heart disease, but over 10 years ago (2004–2014 and 1997–2006 respectively). Global burden showed the most recent citation bursts in the years 2019–2024 (strength = 7.84).

#### 3.6.3 Keyword clustering

**FIGURE 7:**
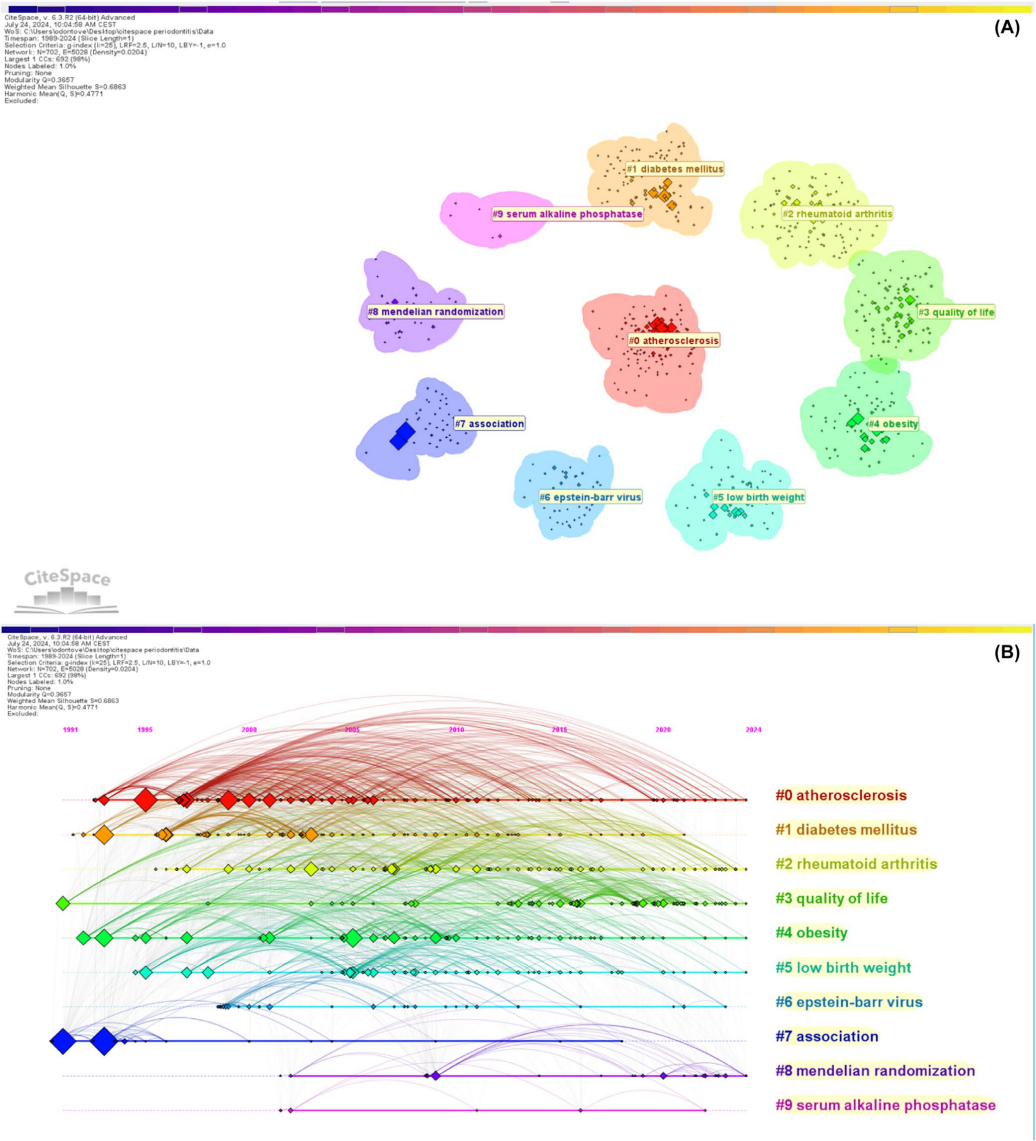
(A) Clustering arrangement of keywords; (B) occurrence over the years of the clustered keywords.

Ten different clusters were obtained after the extraction of cluster labels from terms associated with the titles of articles. Figure 7A shows the clustering arrangement of keywords and Figure 7B includes their occurrences over the years. The outcomes of the clustering showed a modularity Q = 0.3657 and a weighted mean silhouette S = 0.6863, indicating a well‐organized network and a satisfactory clustering arrangement. Among the cluster labels, there were “atherosclerosis,” “diabetes mellitus,” “rheumatoid arthritis,” “quality of life,” “obesity,” “low birth weight,” and “association.”

## 4 DISCUSSION

The objective of the present study was to analyze the bibliometric mapping of observational studies and research trends on the association between periodontitis and systemic conditions. Moreover, it was analyzed a bibliometric design, and the most impactful authors, articles, institutions, and countries who contributed to the development of the association between periodontitis and systemic diseases.

This study's results showed a progressive significant increase over the analyzed period in the number of publications of observational studies linking periodontitis to systemic conditions. More specifically, almost half of the total publications date back to the last 5 years, and the peak of publication was reached in 2023. These data suggest that the analysis of the impact of periodontitis on systemic health (or vice versa) is still in development, and the trend seems to indicate that in the coming years, the number of publications will be even higher. Furthermore, although there has been a steady rise in studies about the connection between systemic conditions and periodontitis throughout the years, there was a decline in papers during the 3 years 2017–2019, with a substantial uptick in the years that followed. It should be noted that most of the top ten articles were published over 10 years ago, which may indicate that some recent articles may not have had the time to be sufficiently quoted to be classified. One possible explanation for this fluctuating trend could be the COVID‐19 pandemic spread that significantly impacted the volume of publications during the emergency.^47^, ^48^ Moreover, in agreement with some recent reports,^49^, ^50^, ^51^ the present results evidenced an upward trend in the number of annual publications related to periodontitis. This could be due to the increase of even more multidisciplinary consensus reports and joint workshops on the association between periodontal diseases and various systemic diseases such as cardiovascular diseases, diabetes mellitus, and respiratory diseases,^52^, ^53^, ^54^ which, together with low‐quality oral hygiene,^55^ may have helped raise awareness in the international scientific community.

Furthermore, a large range of international journals have been published on this topic. Specifically, it was found that the most influential journals in terms of citations were mainly internationally recognized leading journals in the field of periodontology, such as *The Journal of Periodontology*, *The Journal of Clinical Periodontology*, *The Journal of Dental Research*, *Periodontology 2000*, and *The Journal of Periodontal Research*, respectively. However, it should be noted that a substantial contribution also came from medical journals, including Lancet, PLOS ONE, and the New England Journal of Medicine and Circulation. This may be explained by the multidisciplinary nature of the association among the main systemic diseases and periodontitis and by the nature of the disease, which includes a multifactorial etiology.^1^, ^2^, ^56^, ^57^, ^58^, ^59^ A putative link between periodontitis and several systemic diseases could be based on periodontitis, with its chronic burden of periodontal bacteria, which could stimulate oxidative stress conditions and nitric oxide (NO) release, such as cardiovascular diseases and diabetes and may have led to the high production of CRP levels, which in turn could stimulate serum and salivary inflammatory mediators to protect cells from tissue damage because of oxidative stress^60^, ^61^, ^62^ and from specific genes.^63^ Also, some studies showed that periodontitis is positively associated with impaired salivary NO levels.^64^, ^65^ NO can be produced in the gingival tissues as part of the oral unspecific salivary antibacterial defense against anaerobic periodontopathogens bacteria.^66^, ^67^, ^68^, ^69^, ^70^, ^71^ However, there is no consensus about the effects of NO levels during periodontitis. Some of them showed reported lower salivary levels of NO in periodontitis patients.^66^, ^72^ This discrepancy may be because some of the patients enrolled in these studies were smokers, and smoking may increase salivary NO levels.^73^

Regarding countries and institutions, the most impactful nations for the number of published articles were the USA, China, and Brazil, while Harvard University (USA) and Karolinska University (Sweden) were the most contributing institutions, followed by University of London (UK), Universidade de Sao Paulo (Brazil), and Chung Shan Medical University (Taiwan). The larger scientific population could explain these significant contributions, such as active researchers, information access, and financial resources.^38^, ^74^, ^75^ For example, it was shown that each country's public health system is fundamental in the prevention and treatment of periodontitis due to the advancement of science and the increasing of publications citation impact.^76^ In this regard, China and the USA resulted among the nations with the highest percentage of funded publications, with China reaching a peak of 87%.^77^ These results are in agreement with previous data showing that the USA had had the majority of the 100 most‐cited publications (51 articles; 12 648 citations) in the field of periodontology.^78^

Moreover, the present results evidenced that WoS categories related to general medicine, public health, multidisciplinary sciences, endocrinology, immunology, and rheumatology, and vascular diseases were the most significantly correlated with periodontitis, contributing the central role of periodontitis among stomatognathic diseases linked with the systemic ones. Therefore, these data could suggest the growing interest of the medical community in the impact of periodontal diseases on systemic health and management of patients. This is confirmed by the recent release of new clinical recommendations by important international scientific associations, among which the EFP, the World Heart Federation (WHF), the European arm of the World Organization of Family Doctors (WONCA Europe), the Italian Society of Diabetology (SID), Italian Society of Periodontology and Implantology (SIdP), and the Italian Association of Clinical Diabetologists (AMD)^52^, ^53^, ^54^ which developed clinical recommendations and general guidelines for oral healthcare professionals, general doctors and other medical health professions. With the emergence of personalized medicine, the medical community is trying to identify specific factors that can influence individual health^79^, ^80^, ^81^; in this regard, periodontitis was shown as a condition with the main systemic implications and is therefore increasingly included in holistic and personalized approaches to health. According to the MeSH classification, a progressively greater number of publications stented out for most of the sub‐branches related to diseases and conditions. Moreover, of a total of 1519 articles, 1159 studies belonged to the categories (in order) Nutritional & Metabolic Diseases, Cardiovascular Diseases, Female Urogenital Diseases & Pregnancy Complications and Musculoskeletal Diseases. The results derived from keyword citation bursts and clustering also revealed that pregnancy‐related conditions and metabolic and cardiovascular diseases were the most analyzed conditions related to periodontitis. These conditions are those for which there is the greatest scientific evidence.^26^, ^27^, ^28^, ^29^ In this regard, a recent international consensus has evidenced that obstructive sleep apnea, diabetes, chronic obstructive pulmonary disease, and COVID‐19 complications are all independently linked to periodontitis.^52^ Many mechanisms sustaining these associations have been reported. It was evidenced that patients with periodontitis show increased levels of inflammatory mediators related to atherosclerosis, such as C‐reactive protein (CRP) and pro‐thrombotic factors,^82^ increased levels of serum antibodies that react with cardiovascular tissue antigens,^83^ and higher levels of dyslipidemia and oxidative stress.^84^, ^85^ Individuals with both diabetes and periodontitis reported increased bacteremia 80, vascular inflammation 81, and systemic inflammation.^86^, ^87^, ^88^ However, from the sub‐branches classification results, it can be deduced that many articles are also available on other categories of systemic diseases, such as kidney diseases, rheumatoid arthritis, osteoarthritis, cognitive disorders, pulmonary diseases, viral infections, and gastrointestinal disorders. It should also be taken into account that this study is limited to the evaluation of observational studies only. Therefore, given the considerable quantity of articles and the growing publishing trend of this category of studies, a more comprehensively large cohort studies on this topic could be performed in the near future to evaluate the quality of the available evidence and provide more unanimous indications to the international community.

However, after interpreting the results of the present study, some limitations should be discussed. One regards this study's objective to assess the evidence of association between periodontitis and systemic diseases from observational studies. Due to the pauperity of studies on the topic, it was decided that this first analysis should focus only on observational studies. Including the interventional studies may be useful for an even more detailed analysis of the literature trend and the main contributors to its development. Furthermore, it is important to specify that the analysis carried out could not discern the type of association, i.e., whether positive or negative, between periodontitis and systemic diseases evaluated, thus underlining the limitations represented by bibliometric analyses that allow a quantitative evaluation but sometimes, not a qualitative analysis of the available literature.

## 5 CONCLUSION

The present bibliometric and visual study evidenced a growing trend of observational studies evaluating the association between periodontitis and systemic diseases, highlighting the negative impact of periodontitis on many systemic conditions, mainly nutritional and cardiovascular diseases, and a rising translational interest in this relationship in the scientific community. Furthermore, it has been demonstrated that periodontitis patients, with their low but continuous chronic burden of pathological gingival biofilm and dysregulated host response, represent a critical social and health condition that should be prevented and treated early on a large scale, especially in patients susceptible to the disease.

With a generally growing elderly worldwide population, improved knowledge is essential to enable more comprehensive care, preventive strategies, and optimal oral health for patients with periodontitis. However, further studies with a large sample and prospective design are still awaited to better understand the relationship between periodontitis and systemic diseases.

## AUTHOR CONTRIBUTIONS

Gaetano Isola conceived the research, planned and performed the literature review, and wrote the manuscript. Alessandro Polizzi performed the procedures and wrote the manuscript. Sara Serra and Mattia Boato performed the procedures. Anton Sculean validated the results and wrote the manuscript. All the authors gave their final approval and agreed to be accountable for all aspects of the work.

## FUNDING INFORMATION

The study was supported by the “PRIN 2022 Research Projects of National Interest” Italian Minister of the University (project no. 202254FLSB), Principal Investigator Prof. G. Isola, University of Catania, Catania, Italy.

## CONFLICT OF INTEREST STATEMENT

The authors declare no conflict of interest in the present report.

## Supporting information


Tables S1–S2.

## Data Availability

Data are available from the corresponding author upon reasonable request.
