# Supplementary material for: Relationship between periodontitis and systemic diseases: A bibliometric and visual study
Source: Periodontol 2000. 2025 Jan 8;98(1):228–40. doi: 10.1111/prd.12621 (PMC12842847; doi:10.1111/prd.12621)
Supplement: Supplementary file 1 — Tables S1–S2. [file PRD-98-228-s001.docx]

| *No* | *Count* | *Centrality* | *Year* | *Cited journals* |
| --- | --- | --- | --- | --- |
| 1 | 794 | 0.14 | 1989 | Dentistry, Oral Surgery & Medicine |
| 2 | 137 | 0.15 | 1993 | Medicine, General & Internal |
| 3 | 68 | 0.29 | 2003 | Medicine, Research & Experimental |
| 4 | 60 | 0.20 | 1991 | Public, Environmental & Occupational Health |
| 5 | 55 | 0.00 | 2013 | Multidisciplinary Sciences |
| 6 | 45 | 0.44 | 2003 | Immunology |
| 7 | 43 | 0.04 | 2003 | Endocrinology & Metabolism |
| 8 | 36 | 0.00 | 2008 | Rheumatology |
| 9 | 35 | 0.01 | 1997 | Peripheral Vascular Disease |
| 10 | 32 | 0.02 | 2009 | Pharmacology & Pharmacy |

**Supplementary Table 1**. Top 10 WoS categories involved in the publication of articles on the association between periodontitis and systemic diseases.

| *No* | *Count* | *Centrality* | *Year* | *Author* | *Citations* |
| --- | --- | --- | --- | --- | --- |
| 1 | 20 | 0.00 | 2021 | Mahendra, Jaideep | 102 |
| 2 | 17 | 0.00 | 2013 | Gorska, Renata | 189 |
| 3 | 15 | 0.00 | 2021 | Mahendra, Little | 65 |
| 4 | 13 | 0.00 | 2006 | Costa, Fernando Oliveira | 330 |
| 5 | 13 | 0.00 | 2014 | Izumi, Yuichi | 162 |
| 6 | 12 | 0.00 | 2014 | Gomes-Filho, Isaac Suzart | 201 |
| 7 | 11 | 0.00 | 2017 | Chang, Yu-Chao | 362 |
| 8 | 10 | 0.00 | 2019 | D’Aiuto, Francesco | 658 |
| 9 | 10 | 0.00 | 2019 | Miranda Cota, Luis Otavio | 291 |
| 10 | 10 | 0.00 | 2014 | Aoyama, Norio | 91 |

**Supplementary Table 2**. Top 10 authors for the number of published articles on the association between periodontitis and systemic diseases.
